# Abdominal pregnancy: a report of five cases and literature review

**DOI:** 10.3389/fmed.2025.1638015

**Published:** 2025-09-03

**Authors:** Wenxuan Yang, Jianfeng Huang, Yuqing Wang, Huihua Xiang, Daiyu Zhu

**Affiliations:** Hubei Provincial Key Laboratory of Occurrence and Intervention of Rheumatic Diseases, Hubei Provincial Clinical Medical Research Center for Nephropathy, Minda Hospital of Hubei Minzu University, Hubei Minzu University, Enshi, China

**Keywords:** abdominal pregnancy, therapy, diagnosis, hCG, ultrasonography

## Abstract

Abdominal pregnancy is a special type of ectopic pregnancy that require careful evaluation for accurate diagnosis and appropriate management. Detailed clinical presentations, surgical procedures, and histopathological examinations of five cases of abdominal pregnancy are described. Relevant literature is also reviewed to contextualize the findings. These cases highlight the critical importance of precise diagnosis and timely intervention in the management of abdominal pregnancy. This study aims to provide an overview of the diagnosis, treatment, and management strategies of abdominal pregnancy through the presentation of five rare cases.

## 1 Introduction

Ectopic pregnancy occurs in 1–2% of pregnancies worldwide and represents a significant gynecological emergency (1). Abdominal pregnancy, a rare subtype constituting under 1% of ectopic case (2), involves embryonic implantation at extrauterine abdominal sites such as the omentum, major vessels, pouch of Douglas, and peritoneal surface (3–5). Due to nonspecific clinical presentations and diverse implantation locations, these cases frequently evade timely diagnosis. We present five surgically managed abdominal pregnancies at distinct anatomical sites, all demonstrating favorable postoperative outcomes.

## 2 Case presentation

### 2.1 Example 1

**Figure 1:**
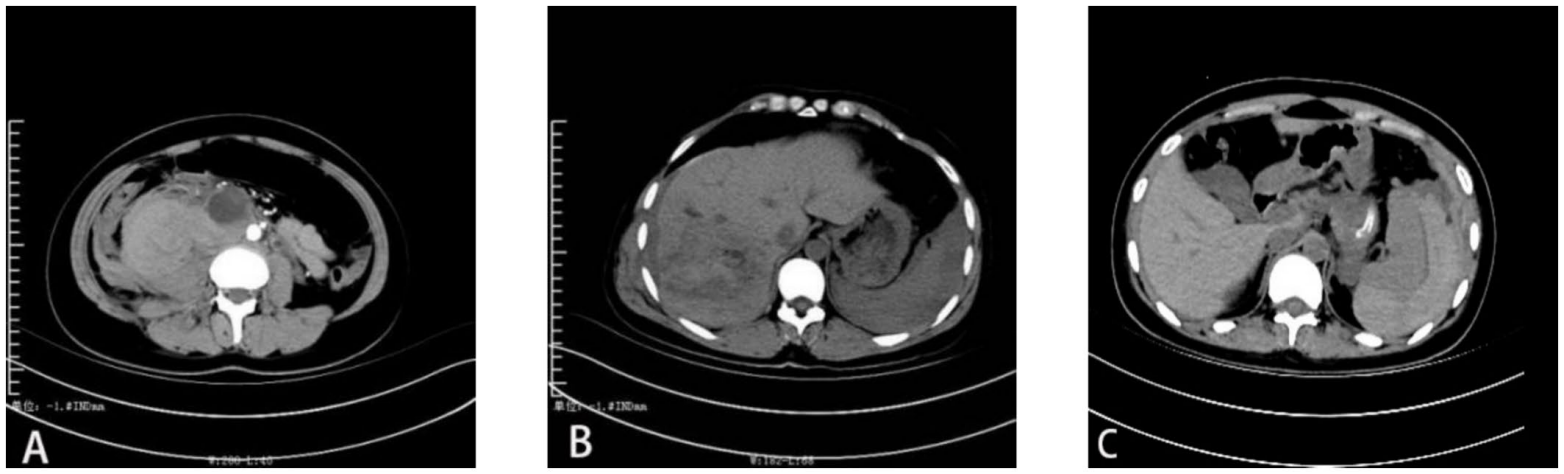
CT images of **(A)** Retroperitoneal pregnancy of the first patient, **(B)** hepatic pregnancy of the second patient, and **(C)** splenic pregnancy of the third patient.

**Figure 2:**
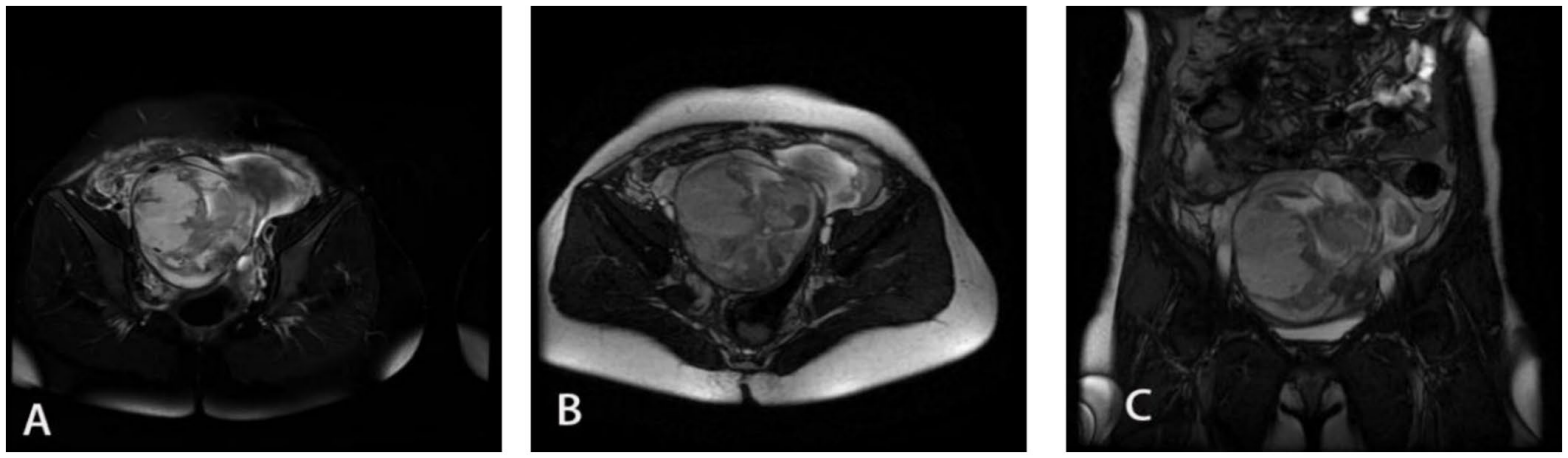
MRI images of Douglas pouch pregnancy of the fourth patient. The three pictures **(A–C)** show different sections of the lesion area of the fourth patient.

**Figure 3:**
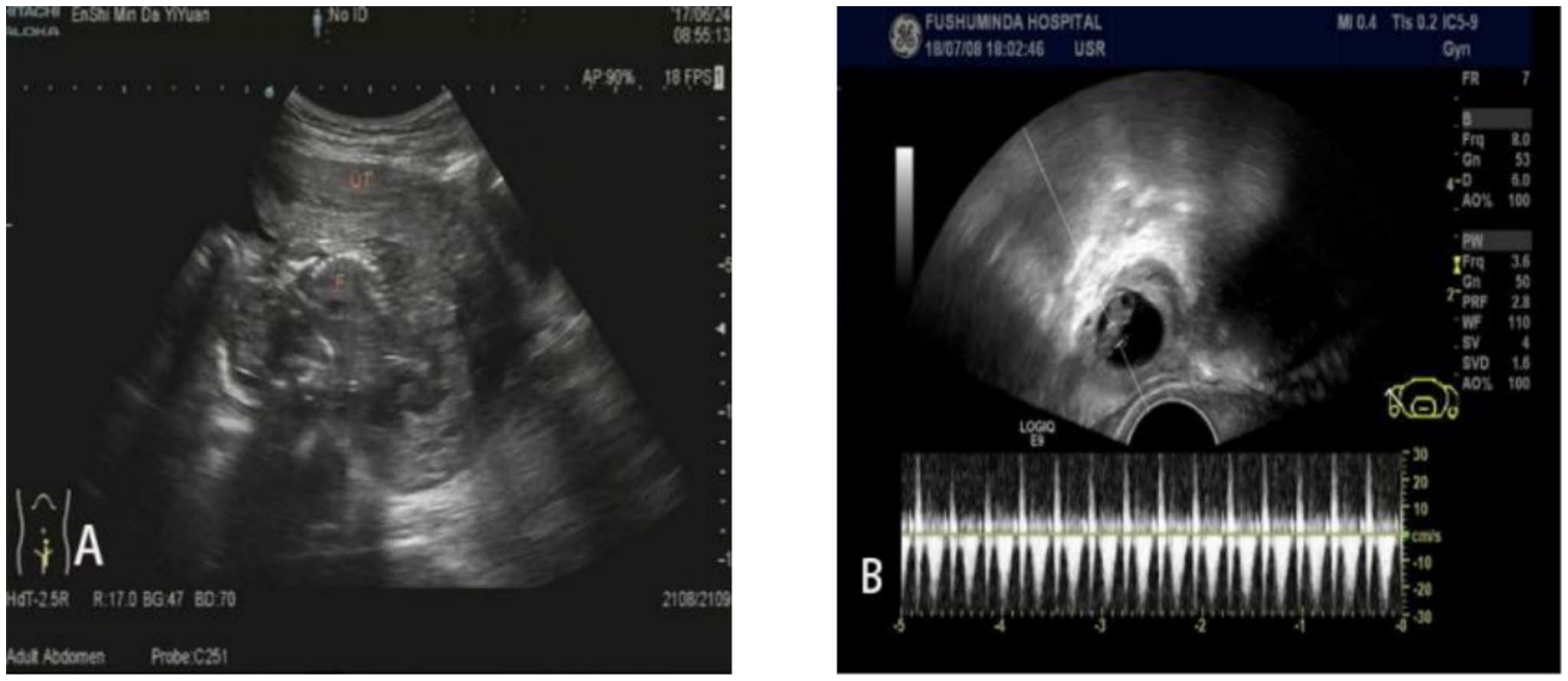
Ultrasonography images of Douglas pouch pregnancy of the fifth patient. The two pictures **(A)** and **(B)** show different sections of the lesion area of the fifth patient.

A 21-year-old female presented to the emergency department with a 1-day history of upper abdominal pain localized below the xiphoid process, which began after dinner and was accompanied by nausea and vomiting of gastric contents. CT imaging revealed acute pancreatitis with pancreatic head hemorrhage, pseudocyst formation, postoperative gallbladder changes, extrahepatic bile duct dilation, and bilateral kidney stones. Laboratory tests showed elevated hCG (14,534.90 mIU/mL) with normal blood amylase (22.0 U/L) and elevated urinary amylase (422.2 U/L). Ultrasound demonstrated normal uterine size with endometrial changes but no intra-or extrauterine pregnancy signs. Initial differential diagnoses included acute pancreatitis and ectopic pregnancy, prompting admission to hepatobiliary-pancreatic surgery. The patient had undergone laparoscopic cholecystectomy in 2008 and cesarean section in 2013. Despite 2 days of conservative management including fasting, gastrointestinal decompression, antibiotics, and pancreatic secretion suppression, follow-up CT showed progressive retroperitoneal hemorrhage of unclear origin. Emergency laparotomy revealed no uterine or adnexal abnormalities but identified a right retroperitoneal hematoma and a 1.5 cm cystic mass between the inferior vena cava and abdominal aorta containing white flocculent material. Histopathological examination confirmed ruptured retroperitoneal ectopic pregnancy. The patient achieved normalized hCG levels by postoperative day 21 and was discharged following full recovery (see Figures 1–3).

### 2.2 Example 2

A 26-year-old female presented to the emergency department with right upper abdominal pain persisting for 1 h following a traffic accident. She reported profuse sweating and localized pain at the injury site. Her medical history included one induced abortion, with her last menstrual period occurring 34 days prior. Physical examination revealed hypotension (90/60 mmHg), pallor, diffuse abdominal tenderness with rebound tenderness, and diminished bowel sounds. Abdominal ultrasonography and CT demonstrated intra-abdominal hemorrhage and hepatic rupture, while the uterus and adnexa appeared normal. Diagnostic paracentesis yielded non-coagulated blood, confirming the diagnosis of liver rupture and necessitating emergency laparotomy. Intraoperative findings included 2,000 mL of hemoperitoneum, substantial clotting beneath the right hepatic lobe, and a 3 cm × 3 cm cystic lesion exhibiting villous tissue and a gestational sac with active hemorrhage. Urine pregnancy testing and serum hCG quantification (5,271 mIU/mL) supported the provisional diagnosis of ruptured hepatic pregnancy. The lesion and adjacent hepatic tissue were excised for histopathological evaluation, which confirmed hemorrhagic rupture of ectopic hepatic pregnancy. The patient achieved biochemical resolution (normalized hCG) by postoperative day 18 and was subsequently discharged.

### 2.3 Example 3

A 20-year-old female presented to the emergency department with 1 day of generalized abdominal distension and pain, accompanied by cessation of flatus and bowel movements, followed by a syncopal episode. Initial evaluation suggested intestinal obstruction or intra-abdominal hemorrhage as potential causes of abdominal pain, prompting admission to general surgery. The patient reported no history of trauma or pregnancy, with her last menstrual period occurring 31 days prior. Physical examination revealed blood pressure of 100/60 mmHg, diffuse abdominal tenderness with guarding and rebound tenderness, diminished bowel sounds, and no palpable masses. A positive urine pregnancy test prompted further evaluation, revealing hemoglobin of 91 g/L and serum hCG of 5,793 U/L. Pelvic ultrasonography demonstrated fluid accumulation with normal uterine dimensions and no intrauterine or adnexal gestational findings. Diagnostic paracentesis yielded 4 mL of non-clotting blood, while abdominal CT identified splenic rupture with active hemorrhage. The patient underwent emergency laparotomy, which revealed significant hemoperitoneum with no uterine or adnexal pathology. Exploration identified active bleeding from a 3 cm^3^ purple-blue cystic lesion at the splenic lower pole, confirmed as the source of hemorrhage. Splenectomy was performed, with subsequent pathological examination of the resected specimen demonstrating chorionic villi and confirming splenic ectopic pregnancy with rupture. Postoperative hCG levels normalized within 10 days, and the patient was discharged following an uncomplicated recovery.

### 2.4 Example 4

A 33-year-old female admitted with a 21 + 3-week history of amenorrhea, persistent abdominal distension for over half a month, and right lower abdominal heaviness for 4 days. Her symptoms began with unexplained abdominal distension and constipation accompanied by flatulence, which persisted for 6 days despite fluid replacement and laxative treatment at a local clinic. The right lower abdominal discomfort occurred without vaginal bleeding or discharge, prompting referral to our hospital. Ultrasound examination revealed no intrauterine fetal echo but detected a formed fetus with cardiac activity and movement in the abdominal cavity posterior to the right uterus. The patient had no prior pregnancy or trauma history before her last menstrual period 21 + 3 weeks earlier. Physical examination showed a blood pressure of 106/67 mmHg, pallor, lower abdominal fullness, and right lower abdominal tenderness, with the uterine fundus at umbilical level and the fetus displaced toward the right lower quadrant. Laboratory tests indicated severe anemia (hemoglobin 63 g/L) and elevated hCG (62,406.30 mIU/mL). MRI confirmed abdominal pregnancy with minimal uterine and pelvic effusion and a sacral canal cyst. Diagnosed with abdominal pregnancy and anemia, she received leukocyte-depleted red blood cell transfusion and underwent exploratory laparotomy. Intraoperative findings included 500 mL of non-coagulated blood and a duck egg-sized mass adherent to the uterine posterior wall and rectal fascia. The amniotic sac contained a 10 cm fetus, and the placenta was attached to the right broad ligament posterior leaf. Both ovaries appeared normal, though the fallopian tubes showed irregular thickening with fimbrial adhesions to the ipsilateral ovaries. The final diagnosis was Douglas pouch pregnancy. Postoperative hCG normalized by day 13, and the patient was discharged following an uncomplicated recovery.

### 2.5 Example 5

A 33-year-old female admitted to a local hospital with a 41-day history of amenorrhea, 8 days of paroxysmal lower abdominal distension, and 1 day of symptom exacerbation. Serum hCG levels were elevated, and ultrasonography revealed abnormal echoes in the right adnexal region with pelvic effusion. A posterior vaginal fornix puncture yielded 4 mL of dark red, non-clotted blood, prompting emergency laparoscopic exploration. Intraoperative findings included slight thickening and bluish-purple discoloration of the right fallopian tube ampulla without rupture or visible villous tissue. Postoperative treatment with methotrexate (MTX), mifepristone, and traditional Chinese medicine failed to control rising hCG levels, necessitating transfer to our institution. Outpatient ultrasonography demonstrated an 18 × 17 mm anechoic gestational sac in the right adnexa containing a yolk sac, embryonic bud, and primitive cardiac pulsation, leading to admission for suspected ectopic pregnancy. The patient had two prior cesarean deliveries but no other surgical or trauma history, with her last menstrual period occurring 57 days before presentation. Physical examination revealed a blood pressure of 91/62 mmHg, fresh laparoscopic scars with intact sutures in the lower abdomen, and no tenderness or rebound tenderness. Serum hCG measured 9084.40 mIU/mL, supporting diagnoses of ectopic pregnancy, mild anemia, and scarred uterus. Repeat laparoscopic exploration showed normal uterine and left adnexal anatomy, while the right fallopian tube ampulla remained thickened and bluish-purple without rupture or active bleeding. A 25 mm× 25 mm purple-blue mass with surface congestion was identified in the right rectouterine pouch, prompting conversion to laparotomy for embryo removal. Gross examination revealed villi and decidual-like tissue, confirmed by pathological analysis showing trophoblasts, blood clots, and chorionic villi. Despite suboptimal hCG decline, the patient requested discharge on postoperative day 5 after acknowledging the risks (see Table 1).

**Table 1:** Case summary.

Serial number	Age (years)	Clinical manifestations	Medical history	Gestational age	Preoperative hCG	Imaging examinations	Preoperative diagnosis	Current treatment	During the operation
1	21	Abdominal pain	Laparoscopic cholecystectomy in 2008 and cesarean section in 2013	Unknown	14534.90mIU/mL	Ultrasonography: There were no signs of pregnancy in or outside the uterus CT:Retroperitoneal hemorrhage increased	Acute Pancreatitis? Ectopic pregnancy?	Laparotomy exploration	A cystic mass about 1.5 cm between the inferior vena cava and the abdominal aorta
2	26	Abdominal pain	Induced abortiononce once	34 days	Unknown	Ultrasonography & CT: Intra-abdominal hemorrhage, liver rupture and bleeding, and no obvious abnormalities were found in the uterus and adnexa area	Liver rupture	Laparotomy exploration	A large amount of blood clot was observed under the right liver, and there was a 3 cm × 3 cm cystic mass
3	20	Abdominal pain	No trauma or pregnancy history	31 days	5,793 mIU/m	Ultrasonography: There were no signs of pregnancy in or outside the uterus CT: Rupture and hemorrhage of the spleen	Ectopic pregnancy and hemorrhagic shock	Laparotomy exploration	A purple-blue cystic mass approximately 3 cm × 3 cm × 3 cm at the lower pole of the spleen, with a ruptured surface and pulsatile hemorrhage
4	33	Abdominal distension	No prior pregnancy or trauma history	21 weeks	62406.30 mIU /mL	Ultrasonography: No obvious fetal echo was found in the uterus, and a formed fetus could be detected in the abdominal cavity behind the right side of the uterus MRI:Pelvic and abdominal pregnancy	Ectopic pregnancy	Conversion from laparoscopy to laparotomy exploration	A mass about the size of a duck egg and a male fist was adhered to the posterior wall of the uterus and the anterior wall of the rectum
5	33	Abdominal distension	Two cesarean sections but no other surgical or trauma history	57 days	9084.40mIU/mL	Ultrasonography: a gestational sac approximately 18 mm × 17 mm in size could be seen in the right adnexal area without echo, and the yolk sac, embryo bud and primitive cardiac tube pulsation could be observed inside	Ectopic pregnancy, mild anemia, and scarred uterus	Laparotomy exploration	A purple-blue mass of approximately 2.5 cm × 2.5 cm at the right rectouterine depression, with a congested surface

## 3 Discussion

Risk factors for abdominal pregnancy encompass congenital reproductive system malformations, prior ectopic pregnancy, previous fallopian tube surgery, uterine rupture, endometriosis, and pelvic inflammatory disease (6, 7).

In early abdominal pregnancy with an intact gestational sac, symptoms often remain nonspecific. Clinical detection typically occurs incidentally during evaluation for intra-abdominal hemorrhage. Previous studies describe presentations including lower abdominal pain, amenorrhea, and vaginal bleeding (8), with some cases exhibiting nausea and vomiting. Without prompt intervention, this condition may progress to life-threatening hemorrhage, demonstrating approximately eightfold higher mortality than tubal pregnancy (6). Clinicians evaluating abdominal pain patients, particularly those with relevant risk factors, should maintain high suspicion for ectopic pregnancy.

Diagnosing abdominal pregnancy typically requires imaging and pathological examination. Ultrasonography detects the presence of yolk sacs, embryos, and vascular pulsation, while CT and MRI intraoperatively localize the implantation site and assess spatial relationships between the gestational sac and adjacent structure (9, 10). Additionally, because of MRI clearly demonstrates the endometrium-myometrium border and the relationship between the endometrial cavity and the gestational sac (11). It can distinguish abdominal pregnancy from other types of ectopic pregnancies. Gerli et al. (12) established ultrasound diagnostic criteria: (a) absence of intrauterine gestational sac, (b) no definitive tubal dilation or complex adnexal mass, (c) peritoneal separation or intestinal encasement of the gestational sac, and (d) sac mobility with fluctuance upon transvaginal probe pressure. When ectopic pregnancy is suspected but pelvic ultrasound proves inconclusive, the examination should extend to abdominal scanning to identify potential hemorrhage sites or extrauterine implantation. In emergent cases requiring laparotomy or laparoscopy, pathological confirmation of chorionic villi provides definitive diagnosis. The principal diagnostic challenge involves promptly considering abdominal pregnancy in acute abdominal presentations with elevated hCG but no evident intrauterine or tubal implantation. Such cases warrant expanded imaging evaluation of rare sites including retroperitoneum, spleen, and liver. CT or MRI precisely maps the gestational sac location preoperatively; when uncertainty persists, surgical exploration with intraoperative ultrasound guidance optimizes localization. Inadequate assessment risks inappropriate management with consequent life-threatening complications and increased healthcare costs.

The management of abdominal pregnancy involves both pharmacological and surgical interventions. Given the invasive nature of placental tissue and the anatomical complexity of gestational sac localization near vascular structures, multidisciplinary consultation is critical for determining an appropriate treatment strategy. Methotrexate (MTX) remains the primary pharmacological option, acting through gestational trophoblast cells proliferation inhibition, villous destruction, and subsequent embryonic tissue necrosis and absorption. MTX may be administered via ultrasound-guided or laparoscopic intragestational sac injection or as systemic therapy perioperatively (13). Huang et al. (14) documented successful CT-guided local MTX injection in two cases, though drug therapy is contraindicated in patients with unstable gestational sacs, gestational age exceeding 11 weeks, significant hemorrhage, or MTX intolerance (15). Most patients failing conservative management ultimately require surgical intervention, as optimal drug regimens remain undefined. Surgical approaches typically involve laparotomy, occasionally requiring collaborative efforts between surgical and radiological teams (3, 16) to identify bleeding sites and facilitate gestational sac removal. Laparoscopic techniques, while offering advantages such as minimal invasiveness and faster recovery (17), demand substantial surgical expertise in retroperitoneal anatomy and may necessitate conversion to laparotomy in cases of undetected gestational sacs or major vascular injury. However, laparoscopic surgery is safer and leads to greater patient satisfaction compared to laparotomy (18). Recent advancements in laparoscopic technology have established it as the preferred exploratory method. Notably, Le et al. (19) reported successful expectant management, underscoring the importance of individualized treatment strategies incorporating gestational parameters, sac characteristics, adjacent organ involvement, clinical presentation, hCG levels, fetal viability, and patient preferences. Early diagnosis and intervention significantly reduce maternal morbidity and improve clinical outcomes.

All five patients presented with abdominal pain but were ultimately diagnosed with abdominal pregnancy during surgical exploration. Surgical intervention was necessary to promptly control hemorrhage and prevent complications. Laparotomy not only confirmed the diagnosis but also facilitated subsequent treatment planning. Postoperative hCG monitoring proved essential for assessing treatment efficacy and determining the need for chemotherapy.

## 4 Conclusion

The clinical implications of this case are noteworthy. Physicians evaluating patients with abdominal pain and abnormal hCG levels should maintain a high index of suspicion for abdominal pregnancy, particularly when supported by relevant medical history. Imaging modalities such as CT and ultrasonography play a pivotal role in establishing the diagnosis. Postoperative surveillance remains critical for patient recovery and complication prevention, where early intervention and surgical expertise significantly influence outcomes. Further investigation into optimized diagnostic approaches and therapeutic protocols for abdominal pregnancy will enhance clinical management strategies.

## Data Availability

The original contributions presented in the study are included in the article/supplementary material, further inquiries can be directed to the corresponding author.
